# The Commoner Contagious Diseases of Childhood

**Published:** 1913-01

**Authors:** 


The Commoner Contagious Diseases of Childhood.—Herrman, (*N. Y. Med. Journal, August* 17, 1912) discusses the means by which these diseases are usually spread. There is something attractive in the idea of air-borne infection but the evidence is all against it. Air and sunlight quickly destroyed the virulence of the pathogenic micro-organisms. Children with different contagious diseases may be treated in the same ward with impunity if contact is prevented by means of the so-called "boxes" and if certain precautions are observed by the nursing staff at the same time. The danger from rooms previously occupied by persons with contagious disease is much less than is generally believed. In New York City alone over $50,000 is expended annually in disinfection and about forty per cent. of this amount for measles, when practically all sanitarians agree that with this disease at least, no disinfection is necessary. In Providence the local health officer, Doctor Chapin, has for some time ceased disinfecting except in special cases, with no noticeable increase in the morbidity of these diseases. Seven weeks' trial in New York gave a like result. The danger from desquamation has been overestimated. Mild unrecognized "missed" cases are responsible to a great extent for the spread of contagious disease. The importance of the role played by the "carriers" cannot be overestimated. Persons, not things, spread disease. Isolation, disinfection, improved medical, school inspection and special hospitals cannot have a marked influence on the reduction of morbidity. This can be accomplished only by a temporary or permanent immunization against these diseases.

